# CHAPERON*g*: A tool for automated GROMACS-based molecular dynamics simulations and trajectory analyses

**DOI:** 10.1016/j.csbj.2023.09.024

**Published:** 2023-09-28

**Authors:** Abeeb Abiodun Yekeen, Olanrewaju Ayodeji Durojaye, Mukhtar Oluwaseun Idris, Hamdalat Folake Muritala, Rotimi Olusanya Arise

**Affiliations:** a MOE Key Laboratory for Membraneless Organelles and Cellular Dynamics, School of Life Sciences, Division of Life Sciences and Medicine, University of Science and Technology of China, Hefei, Anhui, China; b Department of Chemical Sciences, Coal City University, Emene, Enugu State, Nigeria; c Department of Biochemistry, Faculty of Life Sciences, University of Ilorin, Ilorin, Kwara State, Nigeria

**Keywords:** GROMACS, Umbrella sampling, MD trajectory analysis, Kernel density estimation, Free energy landscape

## Abstract

Molecular dynamics (MD) simulation is a powerful computational tool used in biomolecular studies to investigate the dynamics, energetics, and interactions of a wide range of biological systems at the atomic level. GROMACS is a widely used free and open-source biomolecular MD simulation software recognized for its efficiency, accuracy, and extensive range of simulation options. However, the complexity of setting up, running, and analyzing MD simulations for diverse systems often poses a significant challenge, requiring considerable time, effort, and expertise. Here, we introduce CHAPERON*g*, a tool that automates the GROMACS MD simulation pipelines for protein and protein-ligand systems. CHAPERON*g* also integrates seamlessly with GROMACS modules and third-party tools to provide comprehensive analyses of MD simulation trajectories, offering up to 20 post-simulation processing and trajectory analyses. It also streamlines and automates established pipelines for conducting and analyzing biased MD simulations via the steered MD-umbrella sampling workflow. Thus, CHAPERON*g* makes MD simulations more accessible to beginner GROMACS users whilst empowering experts to focus on data interpretation and other less programmable aspects of MD simulation workflows. CHAPERON*g* is written in Bash and Python, and the source code is freely available at https://github.com/abeebyekeen/CHAPERONg. Detailed documentation and tutorials are available online at dedicated web pages accessible via https://abeebyekeen.com/chaperong-online.

## Highlights

• Setting up and running GROMACS MDS can be complex, repetitive and challenging.
• We introduce CHAPERON*g*, a tool that automates GROMACS-based MD simulations.
• It automates conventional and enhanced sampling simulations for diverse systems.
• CHAPERON*g* also offers automated extensive post-simulation trajectory analyses.
• Thus, CHAPERON*g* simplifies the use of GROMACS for biomolecular simulations.

## Graphical abstract

**Figure:**
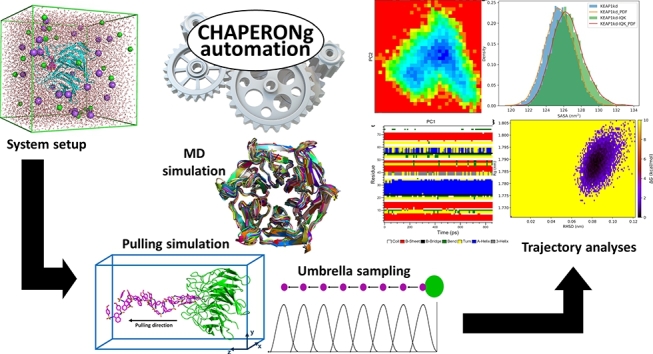

## 1 Introduction

**Fig. 1:**
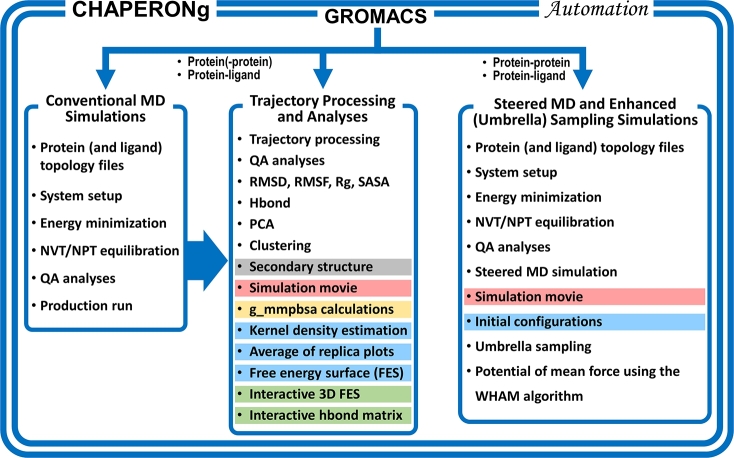
An overview of the workflows and functionalities that CHAPERON*g* offers and automates. These include the entire GROMACS conventional MD simulation (*left*) and the steered MD-umbrella sampling (*right*) workflows, as well as several post-simulation analyses (*middle*). Ligand topologies are generated using parameters obtained from various servers/tools for the CHARMM, AMBER, GROMOS, and OPLS-AA force fields. Functionalities highlighted with various colors indicate those integrated with GROMACS but are offered or largely facilitated by CHAPERON*g* (*blue*) and other third-party tools such as DSSP (*grey*), PyMOL (*red*), g_mmpbsa (*yellow*), MD DaVis (*green*), etc. QA: Quality assurance, RMSD: Root mean square deviation, RMSF: Root mean square fluctuation, Rg: Radius of gyration, SASA: Solvent-accessible surface area, Hbond: Hydrogen bond, WHAM: Weighted histogram analysis method.

Molecular dynamics (MD) simulation is a robust and valuable tool for studying the dynamic behavior, energetics, and interactions of diverse biological systems, including proteins, protein-ligand complexes, nucleic acids, and membrane lipids [1]. These simulations provide insights–in full atomic details and at precise temporal resolutions–into the dynamics, stability, and functional properties of biomolecules, complementing experimental observations and providing guidance for further investigations [2], [3]. GROMACS [4] is a widely used MD simulation software. It is one of the gold standards for biomolecular simulation not only because of its efficiency, accuracy, and extensive range of simulation options but also because it's a free and open-source software with a huge community of users and developers [5], [6]. MD simulation protocols typically consist of three main stages: system preparation, MD production or simulation run, and trajectory analysis (Fig. 1) [7]. While advances in computational power and resources have improved the capabilities of MD simulation tools, setting up and running MD simulations with GROMACS (and other MD simulation codes) still present several challenges [8].

GROMACS is a command-line program and, as with most other popular MD simulation engines, is characterized by limited accessibility to many researchers who, despite possessing the necessary domain knowledge to interpret the relevant computational results, may not be familiar with working on the command line [5], [6]. In addition, setting up and running MD simulations requires a lot of manual tasks, making the process time-consuming, labor-intensive, and error-prone. Since many of these steps are repetitive and programmable, automation tools would greatly minimize manual user interventions, thereby improving efficiency and empowering users (beginners and experts) to focus on other aspects like optimization of parameters, data analysis, and result interpretation [9].

Furthermore, MD simulations typically generate huge amounts of trajectory data, but processing the data into particularly meaningful, relevant, and informative forms often requires programming and data analysis skills [10], [11]. Thus, beginner and intermediate users with limited or no such skills are only able to gain superficial insights from MD output data in spite of MD simulation being a computation-intensive process. The ability to transform simulation data into more interpretable forms and consequently obtain optimally useful information would facilitate the gaining of relevant biological (structural and functional) insights from MD simulations [11].

In order to assuage the aforementioned challenges, several tools integrated into standalone or web-based graphical user interfaces (GUIs) have been developed to automate or simplify various steps or aspects of the GROMACS MD simulation process. The earliest GUI-based programs including GUIMACS [12], jSimMacs [13], and GROMITA [14] that offered some capability to carry out GROMACS MD simulation of protein (only) systems have not been updated for a long time, making them incompatible with recent GROMACS versions [6]. Other GUI-integrated plugins like Dynamics PyMOL plugin [5], [15], Enlighten2 (a PyMOL plugin and Python package) [16], and YAMACS (a YASARA plugin) [6] have such limitations as restrictions to the simulation of specific systems (protein only or protein complexes), support for only select force field(s), lack of trajectory analysis functions, non-trivial installation of dependencies, or the need to learn other software interfaces upon which they depend [16]. Existing web-based interfaces include MDWeb [17] and WebGro [18]; both of which offer MD simulation over limited timescales, and CHARMM-GUI [19]; a toolkit for generating input files for MD simulations using the CHARMM force field. MDWeb does not support the simulation of protein-ligand complexes, and for WebGro, the support is limited to the GROMOS force field. VisualDynamics [8] and BioBB-Wfs [9] are two recent web-based initiatives that also offer MD simulations over limited duration. While they are excellent platforms, they, however, only provide basic analyses of simulation trajectories. They also do not offer advanced simulation workflows (such as biased or enhanced sampling simulations).

In this work, we present CHAPERON*g*, a comprehensive automated pipeline for GROMACS MD simulations and trajectory analyses. CHAPERON*g* is a command-line interface to GROMACS that automates and streamlines the entire MD simulation protocols for protein, protein-ligand, and protein-DNA systems (Fig. 1). It supports ligand topology parameters obtained from popular external parameterization programs for the CHARMM, AMBER, GROMOS, and OPLS-AA force fields. CHAPERON*g* seamlessly integrates with GROMACS modules and third-party tools to enable an extensive automated workflow of up to 20 different post-simulation trajectory and end-point analyses. In addition, it automates the steered MD and umbrella sampling simulations, a biased enhanced simulation protocol often employed to overcome sampling limitations and investigate rare events. Thus, CHAPERON*g* would not only make MD simulation more accessible to beginner GROMACS users but also expand the toolset of experts by facilitating improved efficiency and providing a platform upon which advanced and customized analyses and scripting could be built.

## 2 Methods and code implementation

CHAPERON*g* has been developed using the Bash shell scripting and the Python 3 programming language. The framework and primary modules of the CHAPERON*g* source code were written using Bash shell scripting because GROMACS is a Linux-based software. This allows a seamless GROMACS-CHAPERONg integration and ensures that the only real dependency of CHAPERON*g* is simply a functional GROMACS installation. Thus, the entire MD simulation pipelines can be automatically executed without the need for installation of additional dependencies or software save those required by GROMACS itself.

Other modules of CHAPERON*g* which provide additional and advanced functionalities are written using Python. Various Python libraries are used including Numpy [20], Pandas and Scipy [21]; for data manipulation and numerical and scientific calculations, and Matplotlib [22]; for generating graphical plots and figures. PyMOL [23], ImageMagick, or ffmpeg is used for generating simulation movies. Secondary structure elements are analyzed using DSSP [24]. Xmgrace is used for graph plotting and conversion. The MD DaVis package [11] is used for the construction of hydrogen bond matrices and interactive three-dimensional visualizations of free energy landscapes. Installation of CHAPERON*g* is achieved by simply running the install script provided in the package. To make all features offered by CHAPERON*g* easily accessible to users, an isolated Anaconda Python environment with all needed dependencies can be set up by running a conda setup script also provided in the package.

CHAPERON*g* offers automated GROMACS-based workflows for unbiased conventional MD simulation of protein(-only) and protein complex systems, using established and previously reported protocols [25], [26]. In addition, up to 20 automated analysis types covering system setup and simulation quality assurance analyses as well as post-simulation trajectory analyses are provided. A GROMACS-based workflow for the steered MD and enhanced umbrella sampling simulations for protein complexes are also automated [27], [28], [25]. Automated quality assurance analyses and the WHAM (weighted histogram analysis method)-based free energy calculations [29], [30] are also provided for the biased simulations.

## 3 CHAPERON*g* features and functionalities

CHAPERON*g* can be run in one of two modes of automation depending on the user's choice; either as *full-auto* or *semi-auto*. In the *full-auto* mode, all simulation steps and post-simulation analyses are automatically carried out based on the simulation type and user-provided parameters. This greatly reduces repetitive and tedious manual interventions, and the user is only prompted for inputs in a very few exceptional cases where automatic or pre-defined choices might not be trivial or suitable (e.g. determining the box size of an umbrella sampling simulation). The *semi-auto* mode still has most of the simulation and analyses automated, but the user is prompted more for inputs and confirmation of automatically selected choices to give more flexibility and control over the simulation parameters.

### 3.1 Automated conventional MD simulation

CHAPERON*g* offers automated MD pipelines for various systems, namely protein-only (including protein-protein complexes), protein-ligand complexes, and protein-DNA complexes. For protein-ligand systems, the pipeline recognizes small molecule ligand topologies generated via popular ligand parameterization programs and webservers, including CGenFF (for CHARMM) [31], ACPYPE (for AMBER) [32], PRODRG2 (for GROMOS) [33], and LigParGen (for OPLS-AA) [34]. The automated protocol is organized into 12 major steps, enabling the user to start or resume from any step of the simulation process. The minimum input files required to run CHAPERON*g* are the starting structure and appropriate GROMACS parameter (*.mdp*) files.

#### 3.1.1 System preparation and quality assurance analyses

Once launched, CHAPERON*g* automatically runs through the conversion of the input structure file to the GROMACS format, generation of protein topology (and ligand topology, if applicable), definition of the simulation box, addition of ions to the system, energy minimization and NVT/NPT equilibration steps. For each of these steps, the user has full control over how the system is set up. The system and topology files are automatically updated accordingly, depending on the type of system. Following the energy minimization and equilibration steps, quality assurance analyses–such as plots of the progression of the potential energy, density, temperature, pressure, and other thermodynamic parameters–are run. These enable the user to monitor the convergence of the indices and, hence, the quality of the simulation system.

#### 3.1.2 MD simulation

Following a successful setup of the system, the production MD run proceeds for the duration specified by the user in the corresponding parameter file. CHAPERON*g* also offers the option to call GROMACS to extend a previously completed run, or to resume a terminated simulation. Similar to the system preparation stage, several quality assurance indices including some thermodynamic parameters are analyzed and produced as Xmgrace *.xvg* files as well as publication-quality *.png* figures.

### 3.2 Post-simulation processing and trajectory analyses

CHAPERON*g* provides the capability to carry out up to 20 post-simulation processing and trajectory analyses. These analyses, enabled by modules available in GROMACS, CHAPERON*g*, and other third-party tools, include root mean square deviation (RMSD), root mean square fluctuation (RMSF), radius of gyration (Rg), solvent accessible solvent area (SASA), hydrogen bond (Hbond) analysis, principal component analysis (PCA), secondary structure analysis, clustering analysis, simulation movie, two- and three-dimensional (visualizations of) free energy landscapes (FELs), kernel density estimation (KDE), interactive hydrogen bond matrix, MM-PBSA (Molecular mechanics Poisson–Boltzmann surface area) free energy calculations, and multiple quality assurance analyses. The plots from the analyses are generated as *.xvg* and publication-quality *.png* files. These analyses provide valuable computational metrics for characterizing the stability, folding, conformational changes, interactions and dynamics of biomolecules. For example, they help in the comparison of different MD simulation trajectories, analysis of the impact of mutations or ligand binding, and assessment of the accuracy of simulation models with respect to experimental data.

#### 3.2.1 RMSD, RMSF, Rg and SASA

The RMSD, RMSF, and Rg are three important structural metrics used to characterize the MD simulation of biomolecular systems [35]. The RMSD, Rg, and RMSF are computed in GROMACS by the *gmx rms*, *gmx gyrate*, and *gmx rmsf* modules, respectively. RMSD measures the average distance between the atoms of a structure at an instant of the simulation against the reference starting structure. Thus, it is used to analyze the overall time-dependent structural deviation or similarity between the structures recorded in the trajectory [36], [37]. The RMSD plot of the simulated protein (and that of the ligand in the case of a protein-ligand complex) is generated as *.xvg* files and *.png* figures.

The RMSF, like the RMSD, is a common mobility measure that quantifies the local fluctuations or flexibility within a biomolecule during simulation by measuring the average atomic or residue-level deviations [36]. RMSF provides insights into the dynamic regions of proteins such as flexible loops, and can indicate the importance of specific residues in conformational changes or protein-ligand interactions [38]. Rg is a commonly used measure of the compactness of protein molecules, with smaller Rg values indicating a more compact or folded structure, and larger Rg values suggesting more extended or flexible conformations during the simulation [39].

**Fig. 2:**
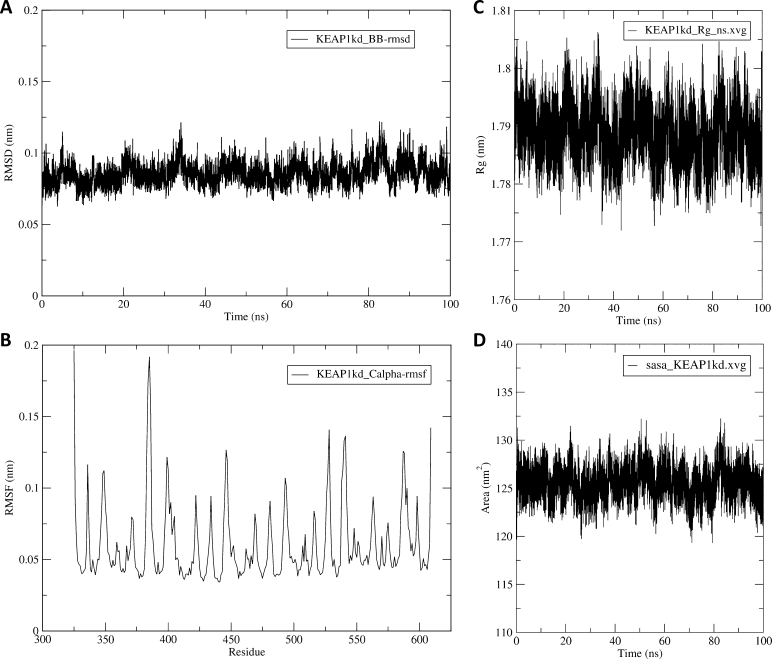
Some trajectory analysis plots generated from the MD simulation of the Kelch domain of the KEAP1 protein (PDB ID 4IQK) as an example. Analyses include the (**A**) Root mean square deviation (RMSD), (**B**) Root mean square fluctuation (RMSF), (**C**) Radius of gyration, and (**D**) Solvent accessible surface area (SASA). For details of the simulation system and setup, see Supplementary Method section 1.

SASA is a metric that provides information about the exposed surface area of a biomolecule that is accessible to the solvent molecules. It is commonly used to investigate protein folding and stability, as well as to characterize the interaction of a protein with the surrounding solvent [40]. SASA is computed in GROMACS by the *gmx sasa* module, which employs the double cubic lattice method [41]–a variant of the “rolling ball” algorithm of Shrake and Rupley [42]. Fig. 2 shows some examples of the automatically generated figures of the RMSD, RMSF, Rg, and SASA plots.

#### 3.2.2 Hydrogen bonding analysis

Hydrogen bond (Hbond) analysis is often used in MD simulations of biomolecules for the investigation and understanding of protein structure, folding, function, and ligand binding as well as other biomolecular interactions [35]. Hbond calculation in GROMACS is carried out using the *gmx hbond* module. Depending on the type of system, the numbers of intra- and inter-molecular Hbonds are calculated and plotted as a function of simulation time. Several other output files, such as the Hbond matrix and index files, are also generated and processed by CHAPERON*g* to parse them as input to other analyses, like the MD Davis-based interactive Hbond matrix calculations.

#### 3.2.3 Principal component analysis

Principal component analysis (PCA) is a statistical technique used to reduce the high-dimensional simulation data–i.e., the coordinates of atoms over time–into a smaller set of orthogonal (principal) components. It helps to visualize the essential dynamics and conformational changes in the trajectory by identifying the most important collective motions in the system [43]. PCA in GROMACS is carried out using the *gmx covar* and *gmx anaeig* modules. The principal components are also processed by CHAPERON*g* and parsed as input for further conformational analyses–e.g., as order parameters for constructing free energy landscapes.

#### 3.2.4 Clustering analysis

**Fig. 3:**
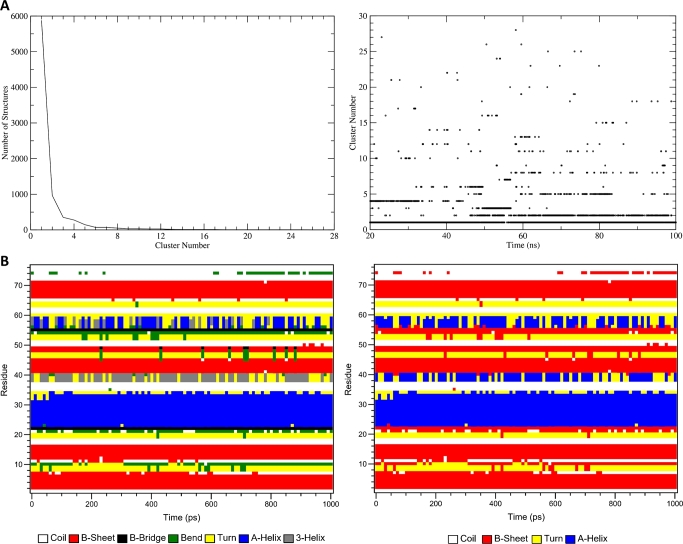
Example output plots of clustering and secondary structure (SS) analyses of MD simulation trajectory. (**A**) Sizes of clusters (*left*) and time-dependent distribution of cluster members (*right*) for the clustering analysis of the ligand-bound Kelch domain of the KEAP1 protein (PDB ID 4IQK) MD simulation trajectory. For details of the simulation system and setup, see Supplementary Method section 3. (**B**) Secondary structure analysis of the human erythrocytic ubiquitin (PDB ID 4GD6) simulation trajectory. Two plots with the seven-SS-type (*left*) and the four-SS-type (*right*) representations are produced. For details of the simulation system and setup, see Supplementary Method section 2.

Clustering in MD simulation is another common technique that is also used to reduce the complexity of trajectory data. It involves grouping similar conformations based on defined structural similarity, enabling the identification of dominant conformational states, dynamics, and transitions [44]. The *gmx cluster* module in GROMACS carries out the analysis, and the automation by CHAPERON*g* maintains the flexibility and array of options it offers. Fig. 3A shows examples of two of the output data plots generated by the analysis.

#### 3.2.5 Secondary structure analysis

Secondary structure (SS) analysis of MD simulation trajectory involves identifying and quantifying the protein secondary structure elements throughout the simulation. The SS analysis in GROMACS is carried out by the *gmx do_dssp* module which relies on the DSSP program [24] for the assignment of SS elements. In addition to the SS analysis plot featuring the default seven SS types assigned by DSSP (see Fig. 3B, *left*), CHAPERON*g* reprocesses the SS elements matrix data to generate a second copy of the plot containing only the four basic SS elements—helices, beta-sheets, turns and coils–as shown in Fig. 3B (*right*). This simplifies the appearance of the plot to aid its visualization and analysis.

#### 3.2.6 Simulation movie

An MD simulation can be summarized into a movie, which is a collection of frames extracted at a specified interval from the trajectory. Simulation movies facilitate the analysis, interpretation, and communication of the simulation results [45]. They provide an animated overview and visual representation of simulations, and can help to easily visualize the motions of regions of interest, such as active sites and pockets, or to observe conformational movements, interactions or displacement of ligands. The minimum requirement for CHAPERON*g* to create a simulation movie is PyMOL, a widely used molecular visualizer. CHAPERON*g* also utilizes either of the ImageMagick *convert* tool or ffmpeg (when either of them is detected on the user's machine) for improved movie quality. Supplementary Files S1 and S2 show two example movies generated by CHAPERON*g* for the example simulations of ubiquitin and ligand-bound KEAP1 Kelch domain, respectively.

#### 3.2.7 Free energy landscapes

**Fig. 4:**
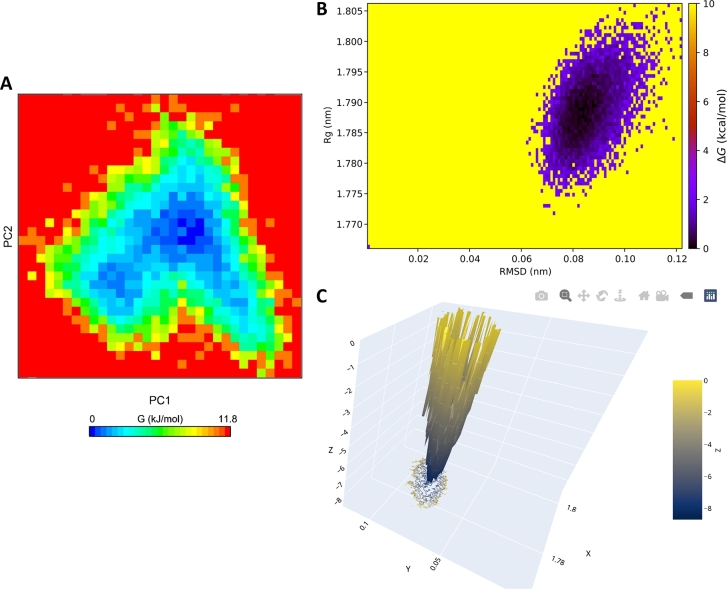
Examples plots of the free energy landscapes (FELs) generated using the free Kelch domain of the KEAP1 protein (PDB ID 4IQK) MD simulation trajectory as an example. (*A*) A 2D plot of the FEL based on principal components generated with gmx sham. (*B*) A CHAPERON*g*-based enhanced 2D plot of the FEL using Rg and RMSD as order parameters. (*C*) An interactive 3D visualization of the Rg-RMSD FEL generated with MD DaVis. For details of the simulation system and setup, see Supplementary Method section 1.

Free energy landscapes (FELs) provide insights into the energetics and stability of different conformational states in MD simulation trajectories. CHAPERON*g* offers three alternative automated ways for the construction of two-dimensional representations of the FEL (Fig. 4). These are enabled by the GROMACS *gmx sham* module for 2D visualizations (Fig. 4A), the CHAPERON*g* energetic landscape module for enhanced 2D visualizations (Fig. 4B), and the MD DaVis tool for interactive 3D visualizations (Fig. 4C). Each of these alternatives requires the user to specify two order parameters for the FEL calculations. Global parameters that describe the state of the system can be used as input, including RMSD, Rg, principal components, fraction of native contacts or number of Hbonds, backbone dihedral angles and configurational distance, etc. [46]. Two preset pairs of order parameters–principal components from a PCA run and the RMSD-Rg pair–are available in CHAPERON*g*. A third option that allows the user to provide other quantities of interest as input is also available.

The FEL calculation by CHAPERON*g* employs a modified version of a previously described method [46], [47]. The relative free energies of states are estimated using Boltzmann inversion as shown in Equation (1). The relative free energy of the most probable state is set to zero while other states are computed to have more positive relative free energies. For all the three approaches, CHAPERON*g* also automates the extraction of the lowest energy structures from the FELs, as well as other FEL-guided structures or frames specified by the user.

(1) $$\Delta G_{i}=-kT\ln\left(\frac{P_{i}(r)}{P_{max}(r)}\right),$$

where *k* is the Boltzmann constant, *T* is the simulation temperature, $P_{i}(r)$ is the probability of the system being in a particular state *i* characterized by some reaction coordinate *r* (quantities of interest) and is obtained from a histogram of the MD data, $P_{max}(r)$ is the probability of the most populated bin (i.e., most probable state), and $\Delta G_{i}$ is the free energy change of the state *i*.

#### 3.2.8 Kernel density estimation

Kernel density estimation (KDE) is a non-parametric technique used to estimate the probability density function (PDF) of a given dataset. This technique utilizes a smooth function, using the Numpy and Scipy Python libraries, CHAPERON*the kernel*, centered at sampled datapoints or bins. The Gaussian kernel is one of the commonly used kernels. Given a sample $x=x_{1},x_{2},\ldots ,x_{n}$ with an unknown density *f* at any given point *x*. The kernel density estimator of the shape of the function *f* is defined as shown in Equation (2).

(2) $${\overset{ˆ}{f}}_{h}(x)=\frac{1}{nh}\sum_{i=1}^{n}K(\frac{x-x_{i}}{h}),$$

where *K* is the kernel (a simple non-negative function such as the Gaussian distribution), and h(>0) is the smoothing bandwidth.

**Fig. 5:**
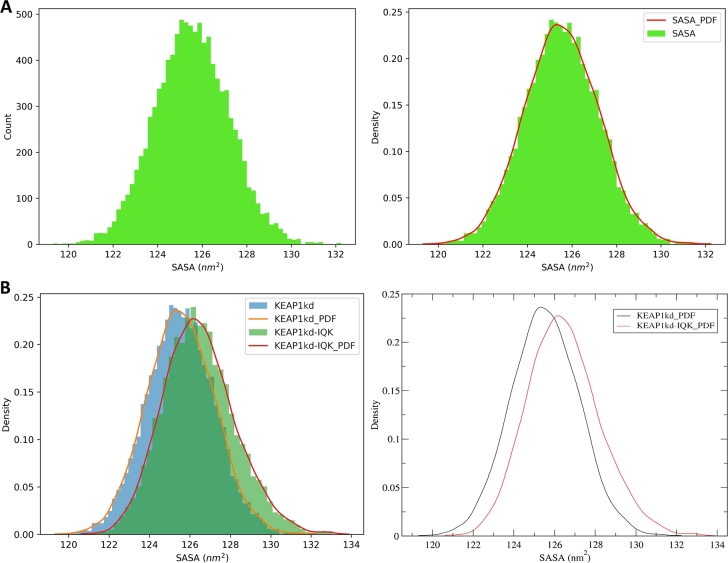
Example CHAPERON*g* kernel density estimation (KDE) plots. (*A*) Histogram (*left*) and KDE (*right*) plots of the SASA data from the example MD simulation trajectory of the KEAP1 Kelch domain. (*B*) Comparative KDE plots of the free KEAP1 Kelch domain protein and the ligand-bound form. Plots are generated as *.png* (*left*) and *.xvg* (*right*) files. For details of the simulation system and setup, see Supplementary Method sections 1 and 3.

Using the Numpy and Scipy Python libraries, CHAPERON*g* automates the kernel density estimation of the PDF for four common MD trajectory data types, including RMSD, Rg, Hbond, and SASA. This estimation can be carried out for single dataset KDE plots (Fig. 5A) as well as for comparative multiple datasets plots (Fig. 5B). The plots are generated as *.xvg* and high-quality *.png* files. Depending on the user's choice, CHAPERON*g* offers automatic and custom selection of the type of bin size estimator, optimal number of histogram bins, and the smoothing bandwidth. The KDE analysis presents a means to gaining further insights into MD simulation trajectories. For instance, the SASA KDE plots shown in Fig. 5 provide a different perspective towards the understanding of the SASA data other than the time-dependent information provided in Fig. 2D.

#### 3.2.9 Interactive hydrogen bond matrix

**Fig. 6:**
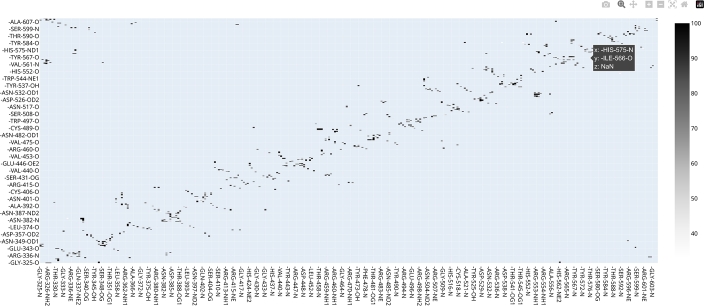
An interactive hydrogen bond matrix generated with MD DaVis using the KEAP1 Kelch domain MD simulation trajectory as an example. For details of the simulation system and setup, see Supplementary Method section 1.

CHAPERON*g* automates the integration of the MD DaVis tool with GROMACS for the construction of a Hbond matrix. To achieve this, CHAPERON*g* prepares a reference (the first structure from the trajectory) and the Hbond list (from the Hbond index file produced by *gmx hbond*). These files are then parsed as input to MD DaVis to produce an interactive *.html* plot (Fig. 6) that gives detailed information about the Hbond contacts recorded in the trajectory [11].

#### 3.2.10 Binding free energy calculations using g_mmpbsa

The g_mmpbsa [48] is a widely used tool that integrates MD simulation with MM-PBSA binding free energy calculation for protein complexes. It also carries out the decomposition of the calculated free energies into contributions per residue [48]. CHAPERON*g* automates and streamlines the workflow for these calculations. Since the original g_mmpbsa is only compatible with GROMACS versions 5.x (or lower) and does not support the more recent and upgraded versions, it has become a common practice for users to install the older GROMACS version as a second copy for use by g_mmpbsa. Thus, the user would need to provide CHAPERON*g* with the path to the appropriate *gmx* executable. However, the g_mmpbsa code has recently been updated by other people [49] and is supposed to support newer GROMACS versions. In this case, there would be no need to specify any path and CHAPERON*g* would automatically call the active GROMACS.

#### 3.2.11 Averaged plot of replica analysis plots

It is a common practice to conduct replica MD simulations of a system, yielding multiple independent trajectories with a higher probability of a wider sampling of the conformational space. Typically, the analysis of the simulations is carried out as means of the replica runs to obtain statistically reliable data, ensure reproducibility, and provide error estimates [50]. CHAPERONg offers a way to automatically generate averaged plots of multiple replica analysis plots, such as the replica plots of the RMSD, Rg, RMSF, SASA, number of hydrogen bonds, or some other user-provided replica plots.

### 3.3 Steered MD and umbrella sampling simulations

**Fig. 7:**
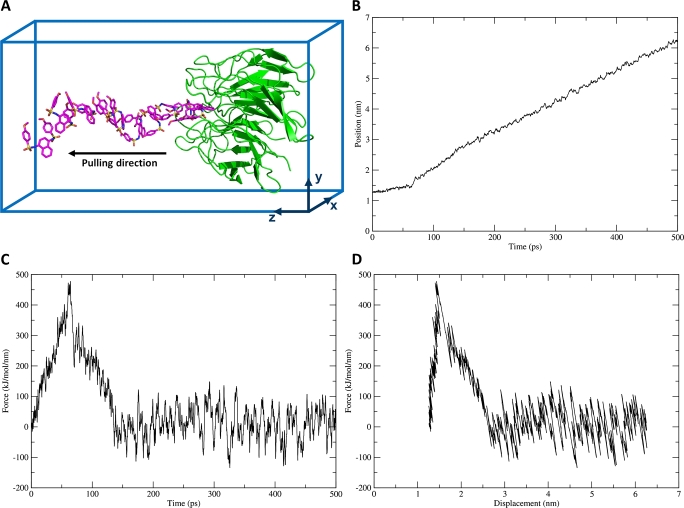
Example steered MD simulation pulling a ligand way from the KEAP1 Kelch domain. (*A*) Illustration of the pulling simulation. The pulled group (ligand) is shown in magenta sticks, and the restrained reference group (receptor) is shown in green cartoon. (*B-D*) Plots of (*B*) pull force against simulation time, (*C*) displacement of the pulled group (the ligand) with time, and (*D*) pull force against the displacement of the pulled group. For details of the simulation system and setup, see Supplementary Method section 4.

**Fig. 8:**
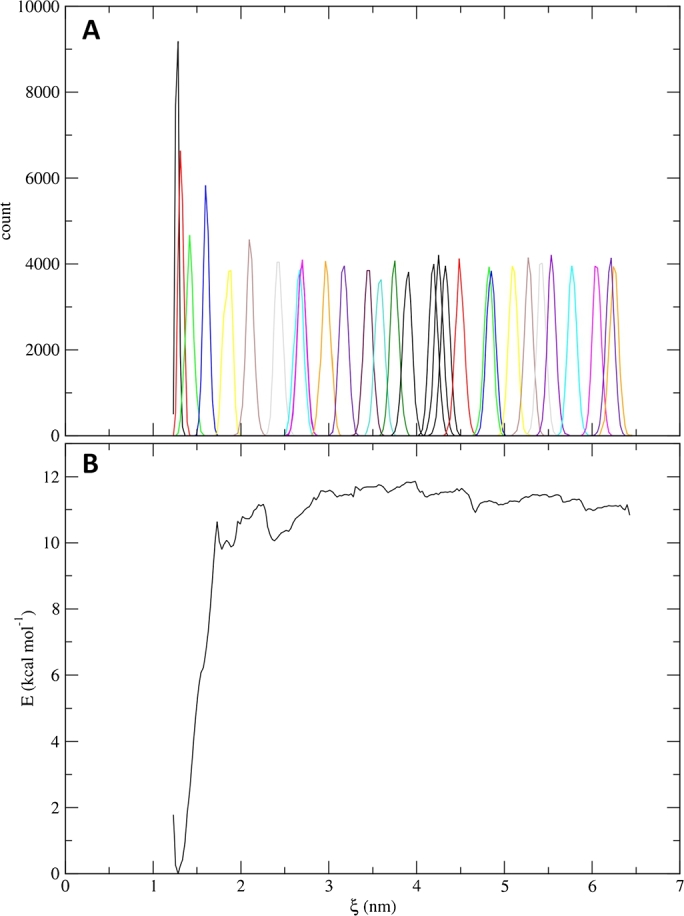
Analysis of an example steered MD and umbrella sampling simulations of the ligand-bound KEAP1 Kelch domain. (**A**) Histograms of the umbrella sampling simulations. (**B**) Potential of mean force (PMF) curve of the ligand unbinding obtained via WHAM calculations. For details of the simulation system and setup, see Supplementary Method section 4.

The steered MD-umbrella sampling simulation workflow is a powerful technique for estimating the free energy of binding for protein complexes [51], [27], [52], and for studying ligand unbinding pathways [28], [53]. This involves a pulling simulation driven by a biasing potential along a given reaction coordinate (Fig. 7). Umbrella sampling simulations are then carried out on a series of configurations in different sampling windows (Fig. 8A). A technique such as the WHAM is finally used to de-bias the system, calculate the potential of mean force (PMF), and consequently, estimate the free energy of binding (Fig. 8B). This entire workflow is streamlined and automated by CHAPERON*g* as briefly described below.

#### 3.3.1 System preparation

Depending on the type of system being simulated, the protein and ligand topologies are generated. Then, a placeholder cubic unit cell is generated and the user is interactively guided to adjust the box and center-of-mass dimensions in an iterative visualize-and-adjust manner (using a molecular visualizer such as PyMOL). This is followed by the solvation, ion adding, energy minimization, and equilibration steps. Several system setup quality assurance analyses are then carried out.

#### 3.3.2 Steered MD simulation and movie

Steered MD simulation involves the pulling apart of the defined pulled and reference groups (illustrated in Fig. 7A). Examples of some of the output files are shown in Fig. 6, including plots of each of the displacement of the pulled group and the pull force against time (Figs. 7B and 7C) and a plot of the pull force against the displacement (Fig. 7D). In addition, a movie of the pulling simulation is also generated. Supplementary File S3 and Supplementary File S4 show example movies for protein-protein and protein-ligand steered MD simulations, respectively. Using the PyMOL interface, the user can customize the renderings in the movie, and then re-run CHAPERON*g* to effect the modifications.

#### 3.3.3 Umbrella sampling

Coordinates are extracted from the steered MD trajectory and the COM distance for each frame is calculated using the *gmx distance* module. Based on user-specified spacing, CHAPERON*g* further uses the distances to identify the starting configurations for the umbrella sampling simulations. Umbrella sampling is then iteratively run for each sampling window.

#### 3.3.4 Potential of mean force and binding energy calculation

Using the WHAM calculations via the *gmx wham* module, the output files from the umbrella sampling simulations are used to compute the PMF and, consequently, the free energy of binding. The plots of the umbrella sampling histograms (Fig. 8A) and the PMF curve (Fig. 8B) are generated as *.png* and *.xvg* files. Also, the binding free energy is calculated and written to a summary file. In a situation where there are windows with insufficient sampling, CHAPERON*g* also offers the possibility to run umbrella sampling for additional user-defined windows.

## 4 Example test cases

Four detailed tutorials using example test cases are available online at dedicated web pages accessible via https://abeebyekeen.com/chaperong-online-tutorials. These include individual tutorials for:

1. Protein-only systems MD simulation.
2. Protein-ligand complex MD simulation.
3. Protein-ligand Umbrella sampling simulation.

In addition, two studies that utilized CHAPERON*g* have recently been published [54], [55] while this article was under review. These works demonstrate the application of CHAPERON*g* for GROMACS MD simulations in drug discovery projects.

## 5 Conclusions

In this work, we have developed CHAPERON*g*, an easy-to-use open-source software that automates the GROMACS MD simulation pipelines for conventional unbiased MD, steered MD, and enhanced umbrella sampling simulations for diverse biomolecular systems. It also offers automated extensive system setup, post-simulation quality assurance analyses, and comprehensive trajectory analyses. Thus, CHAPERON*g* makes MD simulation more accessible to users who have limited experience working with the command line or lack programming skills. It also enables users to gain more insights into MD simulation data by providing an interface that overcomes the technical barriers to processing and analyzing trajectory data. We aim to continuously enhance the usability of CHAPERON*g* based on users' feedback. Future updates would include additional functionalities to expand the capabilities of the software.

## Declaration of Competing Interest

Authors declare no competing interest.

## Data Availability

The CHAPERONg code can be downloaded at https://github.com/abeebyekeen/CHAPERONg. Extensive documentation and detailed tutorials are available online at https://abeebyekeen.com/chaperong-online.

## Appendix A Supplementary material

MMC Supplementary methods. MMC

Video 1 A summary movie of the example 1-ns MD simulation trajectory of ubiquitin in water. Video 1

Video 2 A summary movie of the example 100-ns MD simulation trajectory of ligand-bound KEAP1 kelch domain in water. Video 2

Video 3 A movie of the steered MD simulation of an example protein-protein (BLA-BLIP) complex. Video 3

Video 4 A movie of the steered MD simulation of an example protein-ligand complex (ligand-bound KEAP1 kelch domain). Video 4

## References

[1] Karplus M., McCammon J.A. (2002). Molecular dynamics simulations of biomolecules. Nat Struct Biol.

[2] Hollingsworth S.A., Dror R.O. (2018). Molecular dynamics simulation for all. Neuron.

[3] Hospital A., Goñi J.R., Orozco M., Gelpí J.L. (2015). Molecular dynamics simulations: advances and applications. Adv Appl Bioinform Chem.

[4] Van Der Spoel D., Lindahl E., Hess B., Groenhof G., Mark A.E., Berendsen H.J. (2005). GROMACS: fast, flexible, and free. J Comput Chem.

[5] Makarewicz T., Kaźmierkiewicz R. (2013). Molecular dynamics simulation by GROMACS using GUI plugin for PyMOL. J Chem Inf Model.

[6] Sarkar A., Santoro J., Di Biasi L., Marrafino F., Piotto S. (2022). YAMACS: a graphical interface for GROMACS. Bioinformatics.

[7] Liu H., Jin Y., Ding H. (2023). MDBuilder: a PyMOL plugin for the preparation of molecular dynamics simulations. Brief Bioinform.

[8] Vieira I.H.P., Botelho E.B., de Souza Gomes T.J., Kist R., Caceres R.A., Zanchi F.B. (2023). Visual dynamics: a web application for molecular dynamics simulation using GROMACS. BMC Bioinform.

[9] Bayarri G., Andrio P., Hospital A., Orozco M., Gelpí J.L. (2022). BioExcel Building Blocks Workflows (BioBB-Wfs), an integrated web-based platform for biomolecular simulations. Nucleic Acids Res.

[10] Kagami L.P., das Neves G.M., Timmers L.F.S.M., Caceres R.A., Eifler-Lima V.L. (2020). Geo-Measures: a PyMOL plugin for protein structure ensembles analysis. Comput Biol Chem.

[11] Maity D., Pal D. (2022). MD DaVis:: interactive data visualization of protein molecular dynamics. Bioinformatics.

[12] Kota P. (2007). GUIMACS-a Java based front end for GROMACS. In Silico Biol.

[13] Roopra S., Knapp B., Omasits U., Schreiner W. (2009). jSimMacs for GROMACS: a Java application for advanced molecular dynamics simulations with remote access capability. J Chem Inf Model.

[14] Sellis D., Vlachakis D., Vlassi M. (2009). Gromita: a fully integrated graphical user interface to gromacs 4. Bioinform Biol Insights.

[15] Makarewicz T., Kaźmierkiewicz R. (2016). Improvements in GROMACS plugin for PyMOL including implicit solvent simulations and displaying results of pca analysis. J Mol Model.

[16] Zinovjev K., Van Der Kamp M.W. (2020). Enlighten2: molecular dynamics simulations of protein-ligand systems made accessible. Bioinformatics.

[17] Hospital A., Andrio P., Fenollosa C., Cicin-Sain D., Orozco M., Gelpí J.L. (2012). MDWeb and MDMoby: an integrated web-based platform for molecular dynamics simulations. Bioinformatics.

[18] https://simlab.uams.edu/.

[19] Jo S., Kim T., Iyer V.G., Im W. (2008). CHARMM-GUI: a web-based graphical user interface for CHARMM. J Comput Chem.

[20] Harris C.R., Millman K.J., Van Der Walt S.J., Gommers R., Virtanen P., Cournapeau D. (2020). Array programming with NumPy. Nature.

[21] Virtanen P., Gommers R., Oliphant T.E., Haberland M., Reddy T., Cournapeau D. (2020). Scipy 1.0: fundamental algorithms for scientific computing in python. Nat Methods.

[22] Hunter J.D. (2007). Matplotlib: a 2D graphics environment. Comput Sci Eng.

[23] DeLano W.L. (2002). The PyMOL molecular graphics system. http://www.pymol.org/.

[24] Kabsch W., Sander C. (1983). Dictionary of protein secondary structure: pattern recognition of hydrogen-bonded and geometrical features. Biopolymers.

[25] Lemkul J.A. (2018). From proteins to perturbed Hamiltonians: a suite of tutorials for the GROMACS-2018 molecular simulation package [article v1.0]. LiveCoMS.

[26] Sedzro D.M., Idris M.O., Durojaye O.A., Yekeen A.A., Fadahunsi A.A., Alakanse S.O. (2022). Identifying potential p53-MDM2 interaction antagonists: an integrated approach of pharmacophore-based virtual screening, interaction fingerprinting, MD simulation and DFT studies. ChemistrySelect.

[27] Lemkul J.A., Bevan D.R. (2010). Assessing the stability of Alzheimer's amyloid protofibrils using molecular dynamics. J Phys Chem B.

[28] Sun H., Tian S., Zhou S., Li Y., Li D., Xu L. (2015). Revealing the favorable dissociation pathway of type ii kinase inhibitors via enhanced sampling simulations and two-end-state calculations. Sci Rep.

[29] Kumar S., Rosenberg J.M., Bouzida D., Swendsen R.H., Kollman P.A. (1992). The weighted histogram analysis method for free-energy calculations on biomolecules. I. The method. J Comput Chem.

[30] Souaille M., Roux B. (2001). Extension to the weighted histogram analysis method: combining umbrella sampling with free energy calculations. Comput Phys Commun.

[31] Vanommeslaeghe K., Hatcher E., Acharya C., Kundu S., Zhong S., Shim J. (2010). CHARMM general force field: a force field for drug-like molecules compatible with the CHARMM all-atom additive biological force fields. J Comput Chem.

[32] Sousa da Silva A.W., Vranken W.F. (2012). ACPYPE–antechamber python parser interface. BMC Res Notes.

[33] Van Aalten D.M., Bywater R., Findlay J.B., Hendlich M., Hooft R.W., Vriend G. (1996). PRODRG, a program for generating molecular topologies and unique molecular descriptors from coordinates of small molecules. J Comput-Aided Mol Des.

[34] Dodda L.S., Cabeza de Vaca I., Tirado-Rives J., Jorgensen W.L. (2017). LigParGen web server: an automatic OPLS-AA parameter generator for organic ligands. Nucleic Acids Res.

[35] Ishak S.N.H., Aris S.N.A.M., Halim K.B.A., Ali M.S.M., Leow T.C., Kamarudin N.H.A. (2017). Molecular dynamic simulation of space and earth-grown crystal structures of thermostable T1 lipase Geobacillus zalihae revealed a better structure. Molecules.

[36] Martínez L. (2015). Automatic identification of mobile and rigid substructures in molecular dynamics simulations and fractional structural fluctuation analysis. PLoS ONE.

[37] Sargsyan K., Grauffel C., Lim C. (2017). How molecular size impacts RMSD applications in molecular dynamics simulations. J Chem Theory Comput.

[38] Idris M.O., Yekeen A.A., Alakanse O.S., Durojaye O.A. (2021). Computer-aided screening for potential TMPRSS2 inhibitors: a combination of pharmacophore modeling, molecular docking and molecular dynamics simulation approaches. J Biomol Struct Dyn.

[39] Lobanov M.Y., Bogatyreva N., Galzitskaya O. (2008). Radius of gyration as an indicator of protein structure compactness. Mol Biol.

[40] Savojardo C., Manfredi M., Martelli P.L., Casadio R. (2021). Solvent accessibility of residues undergoing pathogenic variations in humans: from protein structures to protein sequences. Front Mol Biosci.

[41] Eisenhaber F., Lijnzaad P., Argos P., Sander C., Scharf M. (1995). The double cubic lattice method: efficient approaches to numerical integration of surface area and volume and to dot surface contouring of molecular assemblies. J Comput Chem.

[42] Shrake A., Rupley J.A. (1973). Environment and exposure to solvent of protein atoms. Lysozyme and insulin. J Mol Biol.

[43] David C.C., Jacobs D.J. (2014).

[44] Phillips J.L., Colvin M.E., Newsam S. (2011). Validating clustering of molecular dynamics simulations using polymer models. BMC Bioinform.

[45] Lindahl E. (2015).

[46] Tavernelli I., Cotesta S., Di Iorio E.E. (2003). Protein dynamics, thermal stability, and free-energy landscapes: a molecular dynamics investigation. Biophys J.

[47] Papaleo E., Mereghetti P., Fantucci P., Grandori R., De Gioia L. (2009). Free-energy landscape, principal component analysis, and structural clustering to identify representative conformations from molecular dynamics simulations: the myoglobin case. J Mol Graph Model.

[48] Kumari R., Kumar R., Consortium O.S.D.D., Lynn A. (2014). g_mmpbsa–a GROMACS tool for high-throughput MM-PBSA calculations. J Chem Inf Model.

[49] https://github.com/tildeslu/g_mmpbsa.

[50] Knapp B., Ospina L., Deane C.M. (2018). Avoiding false positive conclusions in molecular simulation: the importance of replicas. J Chem Theory Comput.

[51] Grupp B., Lemkul J.A., Gronemeyer T. (2023). An in silico approach to determine inter-subunit affinities in human septin complexes. Cytoskeleton.

[52] Ngo S.T., Vu K.B., Bui L.M., Vu V.V. (2019). Effective estimation of ligand-binding affinity using biased sampling method. ACS Omega.

[53] Tam N.M., Nguyen T.H., Ngan V.T., Tung N.T., Ngo S.T. (2022). Unbinding ligands from SARS-CoV-2 Mpro via umbrella sampling simulations. R Soc Open Sci.

[54] Durojaye O.A., Ejaz U., Uzoeto H.O., Fadahunsi A.A., Opabunmi A.O., Ekpo D.E. (2023). Csc01 shows promise as a potential inhibitor of the oncogenic G13D mutant of KRAS: an in silico approach. Amino Acids.

[55] Durojaye O.A. (2023). Delineation of the CENP-LN sub-complex dissociation mechanism upon multisite phosphorylation during mitosis. J Biomol Struct Dyn.

