# Supplementary material for: CHAPERONg: A tool for automated GROMACS-based molecular dynamics simulations and trajectory analyses
Source: Comput Struct Biotechnol J. 2023 Sep 28;21:4849–58. doi: 10.1016/j.csbj.2023.09.024 (PMC10579869; doi:10.1016/j.csbj.2023.09.024)
Supplement: MMC — Supplementary methods. [file mmc1.pdf]

Supplementary Information

CHAPERON*g*: A tool for automated GROMACS-based molecular dynamics simulations and trajectory analyses

Abeeb Abiodun Yekeen[1,a,*], Olanrewaju Ayodeji Durojaye[1,2], Mukhtar Oluwaseun Idris[1,b], Hamdalat Folake Muritala[3], and Rotimi Olusanya Arise[3,*]

[1] *MOE Key Laboratory for Membraneless Organelles and Cellular Dynamics, School of Life Sciences, Division of Life Sciences & Medicine, University of Science & Technology of China, Hefei, Anhui, China*

[2] *Department of Chemical Sciences, Coal City University, Emene, Enugu State, Nigeria*

[3] *Department of Biochemistry, Faculty of Life Sciences, University of Ilorin, Ilorin, Kwara State, Nigeria*

[*] *Correspondence to: yekeenaa@mail.ustc.edu.cn, arise.ro@unilorin.edu.ng*

[a] Current address: Departments of Biochemistry and Radiation Oncology, University of Texas Southwestern Medical Center, Dallas, TX 75390, USA.

[b] Current address: Biochemistry and Biophysics Department, Oregon State University, College of Science, Corvallis, OR 97331, USA.

Supplementary Methods

1 Molecular dynamics simulation of KEAP1 kelch domain

GROMACS version 2021.6 was used for all the MD simulations described in this work, and the entire pipeline was automated by CHAPERON*g*. The coordinates of the KEAP kelch domain was extracted from the crystal structure file of the protein in complex with a small molecule ligand (PDB code 4IQK). The simulation was performed using the CHARMM36 force field. The system was solvated in a cubic box type using the TIP3P water model. The overall charge of the system was neutralized using sodium and chloride ions. Energy minimization using the steepest descent algorithm was performed for a maximum of 50,000 steps and 1000 KJ/mol/nm. Equilibration was sequentially carried out under the NVT and NPT ensembles at 300 K and 1 bar, each for a duration of 100 ps, using the V-rescale temperature coupling and the Berendsen pressure coupling approaches. Finally, a production run of 100 ns, during which the systems was maintained at 300 K and 1 bar pressure, was executed for the equilibrated system. Several quality assurance and post-simulation trajectory analyses were carried out. For details of the simulation run and output, see the online tutorial at https://abeebyekeen.com/chaperong-tutorial-2-keap1-kelch-domain-in-water.

2 Molecular dynamics simulation of ubiquitin

The coordinates of the human erythrocytic ubiquitin was extracted from its crystal structure file (PDB code 4GD6). The simulation was performed using the OPLS-AA force field. The system was solvated in a dodecahedron box type using the TIP3P water model. The overall charge of the system was neutralized using sodium and chloride ions. Energy minimization using the steepest descent algorithm was performed for a maximum of 50,000 steps and 1000KJ/mol/nm. Equilibration was sequentially carried out under the NVT and NPT ensembles at 300 K and 1 bar, each for a duration of 100 ps, using the V-rescale temperature coupling and the Parrinello-Rahman pressure coupling approaches. A production run of 1 ns was executed with the temperature and pressure maintained at 300 K and 1 bar, respectively. Quality assurance analyses, secondary structure analysis, and generation of simulation movie were carried out. For details of the simulation run and output, see the online tutorial at https://abeebyekeen.com/chaperong-tutorial-1-ubiquitin-in-water.

3 Molecular dynamics simulation of KEAP1-inhibitor complex

The coordinates of the KEAP kelch domain and a co-crystallized small molecule inhibitor were extracted from the crystal structure file of the complex (PDB code 4IQK). The simulation was performed using the CHARMM36 force field, in a cubic box type solvated using the TIP3P water model. Ligand topologies were generated using the CGenFF web server. The overall charge of the system was neutralized using sodium and chloride ions. Energy minimization using the steepest descent algorithm was performed for a maximum of 50,000 steps and 1000KJ/mol/nm. Equilibration was sequentially carried out under the NVT and NPT ensembles at 300 K and 1 bar, each for a duration of 100 ps, using the V-rescale temperature coupling and the Berendsen pressure coupling approaches. Finally, a production run of 100 ns, during which the systems was maintained at 300 K and 1 bar, was executed for the equilibrated system. Several quality assurance and post-simulation trajectory analyses were carried out. For details of the simulation run and output, see the online tutorial at https://abeebyekeen.com/chaperong-tutorial-3-keap1kelchdomain-ligand-complex.

4 Steered MD and umbrella sampling simulations of KEAP1-inhibitor complex

The steered molecular dynamics simulation was carried out over 500 ps. The pulling force was exerted in the z-direction over a distance of 5 nm in a box of size 14 nm. Frames were saved every 1 ps. The ligand topology was generated using the CGenFF server, and the CHARMM36 force field was used for the MD simulations. For the umbrella sampling simulation, a spacing of 0.2 nm was used. This generated a total of 30 initial configurations, capturing the bound to the unbound states of the complex. Within each window, a 100-ps equilibration phase was conducted under the NPT ensemble for a maximum of 50,000 steps. Umbrella sampling simulation was performed in each window for a duration of 2 ns each. The potential mean force (PMF) was calculated using the weighted histogram analysis method (WHAM). The binding free energy (ΔG_{US}) was estimated as the difference between the lowest and highest values along the PMF curve. For details of the simulation run and output, see the online tutorial at https://abeebyekeen.com/chaperong-tutorial-4-keap1kd-ligand-umbrella-sampling.
